# Structure-guided screening of FAR-1 antagonists with multi-stage anthelmintic activity

**DOI:** 10.1128/aac.01226-25

**Published:** 2026-04-15

**Authors:** Juan Wen, Wenmin Qi, Jinwei Lv, Yu Lin, Fei Shen, Yu Li, Ziding Zhang, Lihua Xiao, Yaoyu Feng, Dongjuan Yuan

**Affiliations:** 1 State Key Laboratory of Animal Disease Control and Prevention, Center for Emerging and Zoonotic Diseases, College of Veterinary Medicine, South China Agricultural University 12526, Guangzhou, Guangdong, China; 2 College of Biological Sciences, China Agricultural University 34752, Beijing, China; The Children's Hospital of Philadelphia, Philadelphia, Pennsylvania, USA

**Keywords:** FAR-1, antagonist, anthelmintic, *Nippostrongylus brasiliensis*, *Angiostrongylus cantonensis*

## Abstract

Fatty acid and retinol binding proteins (FARs) are nematode-specific proteins that orchestrate lipid metabolism, development, and host immune response. Here, the antagonists of *Nippostrongylus brasiliensis* FAR-1 (*Nb*FAR-1) were identified through integrating virtual screening, fluorescent ligand binding assay, and *in vitro* egg hatching assays. An *in vivo* mouse model was employed to evaluate anthelmintic efficacy against intestine-parasitized *N. brasiliensis* and brain-parasitized *Angiostrongylus cantonensis*. Forty-eight candidates were selected by virtual screening, six of them showed more than 40% antagonism to *Nb*FAR-1 by fluorescent ligand-competition binding assay and suppressed *N. brasiliensis* egg hatching by 40%–80% at 20 μM. In mice, E002-0872 and 4340-0245 reduced intestinal *N. brasiliensis* burdens by 61.74% and 62.15%, respectively, and ameliorated intestinal damages. 4340-0245 reduced cerebral *A. cantonensis* burdens by 52.73% and alleviated meningeal bleeding and neurological signs. Moreover, treatment with 4340-0245 at 15 mg/kg shortened the body length of female worms, consistent with the higher *far-1* expression in females. Alanine scanning showed I95 of *Nb*FAR-1 as a key residue for binding fatty acid, retinol, and 4340-0245. Intraperitoneal administration of 4340-0245 at 50 mg/kg did not cause any significant toxic effects, whereas 15 mg/kg resulted in a plasma *C*_max_ of 56,473 ng/mL at 10 min and a half-life of ~7 h. These data provide evidence that FAR-1 is a promising target for developing anthelmintic drugs.

## INTRODUCTION

Nematode infections pose a significant threat to human health and animal welfare, impacting global public health and the cultivation of economic animals. Nevertheless, the restricted selection of anthelmintic drugs available, in conjunction with their deleterious side effects and the mounting concern regarding the emergence of drug resistance (1), underscores the pressing imperative to pinpoint novel therapeutic targets for the management of helminthiasis. Recent advances have highlighted the importance of specific genes in the growth and development of nematodes. This has opened up opportunities for targeted interventions that could disrupt parasitic life cycles while minimizing host toxicity (2–6).

Fatty acid and retinol binding proteins (FARs) are regarded as lipid-binding proteins that are unique to nematodes and possess the typical *Gp*FAR-1 domain. To date, only a few *far*-like genes have been identified in certain symbiotic bacteria with nematodes (7). The FAR-1 protein is crucial for nematode development and is predominantly found in the epidermis, intestines, eggs, and ovaries of female worms (8–10). In our previous work, interfering with the expression of the *far-1* gene in *Nippostrongylus brasiliensis* led to a reduction in body size of L3 larvae and adult worms, as well as a decrease in egg hatching rates and larval development efficiency (11). Similarly, the silencing of the *far-1* gene in *Aphelenchoides ritzemabosi* results in a significant decline in its reproductive capacity, with a reduction of over 70% (12). Furthermore, the expression level of *far-1* in the highly pathogenic strain Rs-C of *Radopholus similis* is notably higher than that in the low pathogenic strain Rs-P (9). Silencing the *far-1* gene expression has been demonstrated to attenuate the pathogenicity of plant-parasitic nematode (9). Additionally, the silencing of the *Abfar-1* gene in *Aphelenchoides besseyi* or the *Gpfar-1* gene in *Globodera pallida* has been shown to result in a 40%–60% decrease in the nematodes’ infection efficiency (13, 14). Consequently, the FAR-1 protein, which is specific to nematodes, is indispensable for both the development and pathogenicity of these organisms.

The nematode-specific FAR-1 protein has emerged as a promising target for the development of novel, low-toxicity anthelmintic drugs. *N. brasiliensis*, soil-transmitted intestinal nematode, is also named “rat hookworm” and regarded as a model for studying anthelmintic drug and host immunopathology (15–17). *N. brasiliensis* has been maintained in the laboratory conditions for over two decades (18). In addition, to broaden the spectrum of the antagonists’ anthelmintic activity across nematode species parasitized in different anatomical niches, *Angiostrongylus cantonensis* residing in the brain was employed. *A. cantonensis*, biological-transmitted zoonotic nematode, parasitizes in the brain of mice and mammals (19) and causes severe damage and lesions of host brain (20). The objective of this study is to characterize the novel FAR-1 antagonists with anthelmintic effects derived from extensive compound databases by virtual screening, fluorescent ligand binding assay, *in vitro* egg hatching tests, and *in vivo* mouse model. Furthermore, the key amino acid sites within the ligand binding pocket of FAR-1 were identified for further structural optimization. This study provides a foundation for the development of novel anthelmintic drugs.

## MATERIALS AND METHODS

### Worm collection and culture

*N. brasiliensis* and *A. cantonensis* were maintained in our laboratory. The worm collection and culture were performed as described in our previous studies (11, 19).

### Molecular docking and virtual screening

The three-dimensional structure of the target protein FAR-1 was constructed through homology modeling using the Swiss-Model server (20), with the holo conformation of *Necator americanus* FAR-1 (PDB ID: 4XCP) serving as the structural template to preserve the native ligand-binding topology (21). Subsequent protein preparation was performed using Schrödinger’s Protein Preparation Wizard workflow (New York, USA), which included hydrogen atom addition, hydrogen-bond network optimization, bond order assignment, and reconstruction of incomplete regions via Prime. Protonation states of ionizable residues were determined at physiological pH 7.4 through local microenvironment analysis, followed by constrained energy minimization (OPLS3 force field) converging at 0.30 Å heavy-atom root mean square deviation (RMSD). For ligand preparation, a combinatorial library was assembled from the Chemdiv-core collection (approximately 215,000 compounds) and a specialized antimalarial database (~15,000 compounds) (22). All compounds underwent standardized preparation through the LigPrep module. Receptor grid generation focused explicitly on the ligand-binding pocket (plm_201), corresponding to the endogenous substrate site in the *Na*FAR-1 template. A 12 × 12 × 12 Å³ grid box was defined to encapsulate the binding cavity, with default van der Waals scaling factors applied to accommodate atomic flexibility.

Virtual screening was performed using a hierarchical cascade approach, whereby the full compound library was initially processed through Glide’s HTVS mode with flexible ligand sampling. The top 10% of HTVS hits (approximately 21,500 compounds) were prioritized by GlideScore and progressed to Standard Precision (SP) docking for refined pose prediction. From the SP results, the top 10% of scoring compounds (approximately 2,150) were advanced to the stage of hit selection. The identification of the final candidate was based on three criteria: (i) quantitative assessment of docking score (GlideScore), (ii) visual validation of binding poses within the ligand-binding site to confirm interactions with catalytic residues, and (iii) chemical diversity analysis. This multi-parametric approach yielded 75 prioritized candidates for experimental validation.

### Fluorescent ligand competition binding assay

Forty-eight compounds (Topscience, China) and 11-(dansylamino) undecanoic acid (DAUDA) (Sigma-Aldrich, Germany) were dissolved in dimethylsulfoxide (DMSO) to a concentration of 1 mM, respectively. The reaction was conducted in a 96-well black enzyme-linked immunosorbent assay plate, with samples divided into distinct groups. The experimental groups were as follows: control group (10 μg *Nb*FAR-1 protein), DAUDA group (10 µL of 1 mM DAUDA), DAUDA and *N. brasiliensis* FAR-1 protein group (10 μg of *Nb*FAR-1 protein and 10 µL of 1 mM DAUDA), and experimental groups (10 μg of *Nb*FAR-1, 10 µL of 1 mM DAUDA, and compounds). The excitation wavelength was set at 345 nm, and fluorescence values were detected within the range of 380–700 nm, with values recorded at 5 nm intervals.

### Methyl thiazolyl tetrazolium method

The effect of the compounds on cell growth was evaluated using HEK-293T cells. The cells were divided into three groups: the DMSO group, the albendazole group, and the 10 μM compound group. The culture medium in each well was replaced with 200 μL of serum-free medium after 24 h of cultivation. Subsequently, 20 μL of MTT chromogenic reagent (Solarbio, China) was added to each well, followed by incubation at 37°C for 1–3 h. The absorbances were measured at a wavelength of 490 nm.

### Egg hatching of *N. brasiliensis*

Female adults were retrieved from the small intestines of SD rats at dpi 11. These worms were then placed into a culture medium and incubated at 37°C for 1 h to allow them to lay eggs. The eggs were collected and transferred to 96-well culture plates after the adult worms were removed. The eggs were divided into several groups: a negative control group (1% DMSO), positive control groups (albendazole at concentration of 10 μM, 20 μM, 40 μM, 80 μM), and compound groups with concentrations of 10 μM, 20 μM, 40 μM, and 80 μM, respectively. The number of first-stage (L1) larvae in each well was counted after incubation for 16 h at 37°C and 5% CO_2_. The hatching rate of the eggs was subsequently calculated.

### Anthelmintic effects of candidate antagonists to *N. brasiliensis*

Twenty-eight BALB/c mice were divided into seven groups after a 3-day acclimatization period. Each mouse was infected with 800 L3 larvae of *N. brasiliensis*. The compound was administered via intraperitoneal injection starting on dpi 0 and continued for 3 consecutive days. The mice were assigned to the following groups: Naive group (uninfected, normal mice), DMSO group (infected mice treated with 10% DMSO from dpi 0 to dpi 3), Albendazole group (infected mice treated with 20 mg/kg albendazole), and compound groups (infected mice treated with 5, 10, 15, and 20 mg/kg of the compound, respectively).

The body weight of the mice was recorded. BALB/c mice infected with *N. brasiliensis* were euthanized on dpi 8. Lung and small intestine tissues were excised from the mice, fixed in 4% paraformaldehyde, and prepared for hematoxylin and eosin (HE) staining and subsequent microscopic observation. The tissues extending from the stomach to the cecum were carefully removed, immersed in pre-warmed phosphate buffered saline (PBS) at 37°C, and longitudinally dissected under a stereomicroscope to collect the worms. The collected worms were washed with PBS buffer, fixed in 4% paraformaldehyde, and examined under a stereomicroscope. The morphologies of the female and male worms were photographed, and their body lengths were measured using Image J software.

### Anthelmintic effects of candidate antagonists to *A. cantonensis*

Thirty BALB/c mice were divided into six groups after a 3-day acclimatization period. Each mouse was infected with 50 L3 larvae of *A. cantonensis*. The compound was administered via intraperitoneal injection starting on dpi 1 and continued for 7 consecutive days. The mice were assigned to the following groups: Naive (uninfected, normal mice), DMSO (infected mice treated with 10% DMSO from dpi 1 to dpi 7), Albendazole (infected mice treated with 20 mg/kg albendazole), and 4340-0245 (infected mice treated with 7.5, 15, and 30 mg/kg of compound 4340-0245).

The body weight of the mice was recorded. The neurological signs of the mice were evaluated using both the Longa scale and the Garcia method. The mice were euthanized and dissected on dpi 15. The number of L4 larvae in the brains was counted, and their morphology was observed. Additionally, brain tissues were collected from the mice for HE staining and subsequent microscopic examination.

### Preparation of *Nb*FAR-1 mutants

The alanine scanning method was employed to mutate amino acid residues on the surface of the ligand-binding pocket of *Nb*FAR-1 protein. Ten prokaryotic expression plasmids harboring point mutations in *Nbfar-1* were constructed by homologous recombination. Using the pET-28a-*Nbfar-1*(WT) plasmid as a template, polymerase chain reaction (PCR) amplification was performed with a pair of primers designed to introduce the specific mutation sites, as listed in Table S1. These plasmids were subsequently transformed into *E. coli* (DE3) competent cells, and expression was induced by the addition of 1 mM isopropyl β-D-thiogalactoside (IPTG) at 16°C for 16 h. The *Nb*FAR-1 mutants were purified and verified by sodium dodecyl sulfate-polyacrylamide gel electrophoresis (SDS-PAGE). A fluorescent ligand binding assay was then conducted to evaluate the binding capabilities of the purified proteins, including the wild-type FAR-1(WT) and the mutants FAR-1(F41A), FAR-1(L45A), FAR-1(L53A), FAR-1(L87A), FAR-1(I95A), FAR-1(F105A), FAR-1(I109A), FAR-1(I110A), FAR-1(L153A), and FAR-1(L160A). The binding affinities of these proteins with 10 μM DAUDA, 10 μM oleic acid, 10 μM retinol, and 10 μM 4340-0245 were assessed.

### Sequence alignment

A total of 13 FAR-1 proteins were retrieved from the UniProt database, including *Wuchereria bancrofti* (A0A3P7DJ73), *Brugia pahangi* (A0A0N4TJG8), *Brugia malayi* (A0A0J9XUY7), *Loa loa* (Q962W7), *Onchocerca ochengi* (Q8WT57), *Thelazia callipaeda* (A0A0N5D8N3), *Acanthocheilonema viteae* (A0A498SJI9), *Rhabditophanes* sp. KR3021(A0A1I8C6P4), *Onchocerca flexuosa* (A0A238C210), *Onchocerca gutturosa* (Q8WT59), *Haemonchus contortus* (C5J3Q7), *Haemonchus placei* (A0A0N4WVP4), *Teladorsagia circumcincta* (A0A2G9T3K2), *Ascaris suum* (F1LEI7), *Caenorhabditis elegans* (P34382), *Steinernema carpocapsae* (A0A4U5MD33), *Diploscapter pachys* (A0A2A2LQU3). Subsequently, sequence alignment of these FAR-1 proteins was performed using MEGA 11 software, and the alignment result was visualized by ESPrint 3.0.

### Molecular docking

The 3D structure of *Nb*FAR-1 was predicted using the AlphaFold3 server (https://alphafoldserver.com/), and the protein structure was visualized using PyMOL-viewer (https://pymol.org/2/). The structural files of DAUDA, retinol, oleic acid, and compound 4340-0245 were downloaded from the PubChem database (https://pubchem.ncbi.nlm.nih.gov/). *Nb*FAR-1(WT) and mutant FAR-1(I95A) were subjected to dehydration and hydrogenation, and the ligands were hydrogenated. The 3D structure of the *Nb*FAR-1 protein and the structural file of the ligands were imported into Autodock Vina software for molecular docking. The conformation with the lowest binding energy was selected. The docking results were then imported into Discovery Studio software for interaction force analysis.

### Acute toxicity test

BALB/c mice of each sex were divided into two groups, with six mice in each. The mice were fasted for 12 h prior to the experiment. For each sex, one group was designed as the control group and received an intraperitoneal injection of the solvent, while another group was administered 50 mg/kg compound 4340-0245 via intraperitoneal injection. The vital signs of the mice were monitored immediately after administration at the following time points: 5 min, 10 min, 30 min, 1 h, 2 h, 3 h, 4 h, 6 h, 8 h, 12 h, and within 14 days. The survival rate and any abnormal behaviors of mice, including convulsion, drowsiness, listlessness, and hair loss, were observed. The body weight was measured daily and continuously recorded for 14 days following administration. Additionally, H&E staining was performed on the hearts, livers, spleens, lungs, and kidneys of the mice in each group after administration.

### Liquid chromatography-tandem mass spectrometry

Five microliters of methanol-water mixture (volume ratio 1:1) was added to 100 μL of mouse plasma, followed by vortexing. Subsequently, 300 μL of methanol was added, and the mixture was centrifuged at 14,000 *g* for 30 min. Two microliters of the supernatant was then used for LC-MS/MS analysis. The chromatographic separation was performed on a Phenomenex C18 column (100 mm × 2.1 mm × 2.6 µm) with an injection volume of 2 μL and a column temperature set at 40°C. The mobile phase consisted of water containing 0.1% formic acid (mobile phase A) and methanol (mobile phase B) at a flow rate of 0.4 mL/min. The gradient elution program was as follows: mobile phase B was 10% from 0.0 min to 0.3 min, increased to 95% from 0.3 min to 0.6 min and maintained from 0.6 min to 2.5 min, then returned to 10% from 2.5 min to 2.6 min and maintained 2.4 min.

The compounds were quantitatively analyzed using positive ion mode detection with an electrospray ionization (ESI) source and multiple reaction monitoring (MRM) mode. The ion pairs used for quantitative and qualitative analyses were *m*/*z* 484.2 → 416.2 and *m*/*z* 484.2 → 123.2, respectively. A systematic methodological validation was conducted for the determination of compound 4340-0245 in plasma samples in accordance with the Chinese Pharmacopoeia 9012 Guidelines for Validation of Quantitative Analytical Method of Biological Samples. The validation parameters included selectivity, standard curve, lower limit of quantification (LLOQ), precision, accuracy, recovery rate, and stability.

### Pharmacokinetics

Thirty male BALB/c mice were divided into six groups. Each animal was intraperitoneally injected with 15 mg/kg of 4340-0245 after fasting for 12 h and water deprivation for 6 h. Blood samples (approximately 0.3 mL) were collected from each group at two different time points: Group a (10 min, 2 h), Group b (20 min, 4 h), Group c (30 min, 6 h), Group d (40 min, 8 h), Group e (50 min, 24 h), and Group f (1 h, 48 h). The plasma was separated by centrifugation at 4°C for 10 min at 2,000 *g* and stored at −80°C. The concentration of 4340-0245 in plasma was detected using the established LC-MS/MS method. Pharmacokinetic, including the area under the curve from time zero to the last measured time point (AUC_0-t_) and from time zero to infinity (AUC_0-∞_), elimination half-life (*t*_1/2_), time to reach peak concentration (*t*_max_), clearance rate (CL/F), and peak concentration (*C*_max_) in BALB/c mice, were calculated using WinNonLin 8.1 software with a non-compartmental model.

## RESULTS

### Library screening and identification of candidate antagonists of *Nb*FAR-1

**Fig 1:**
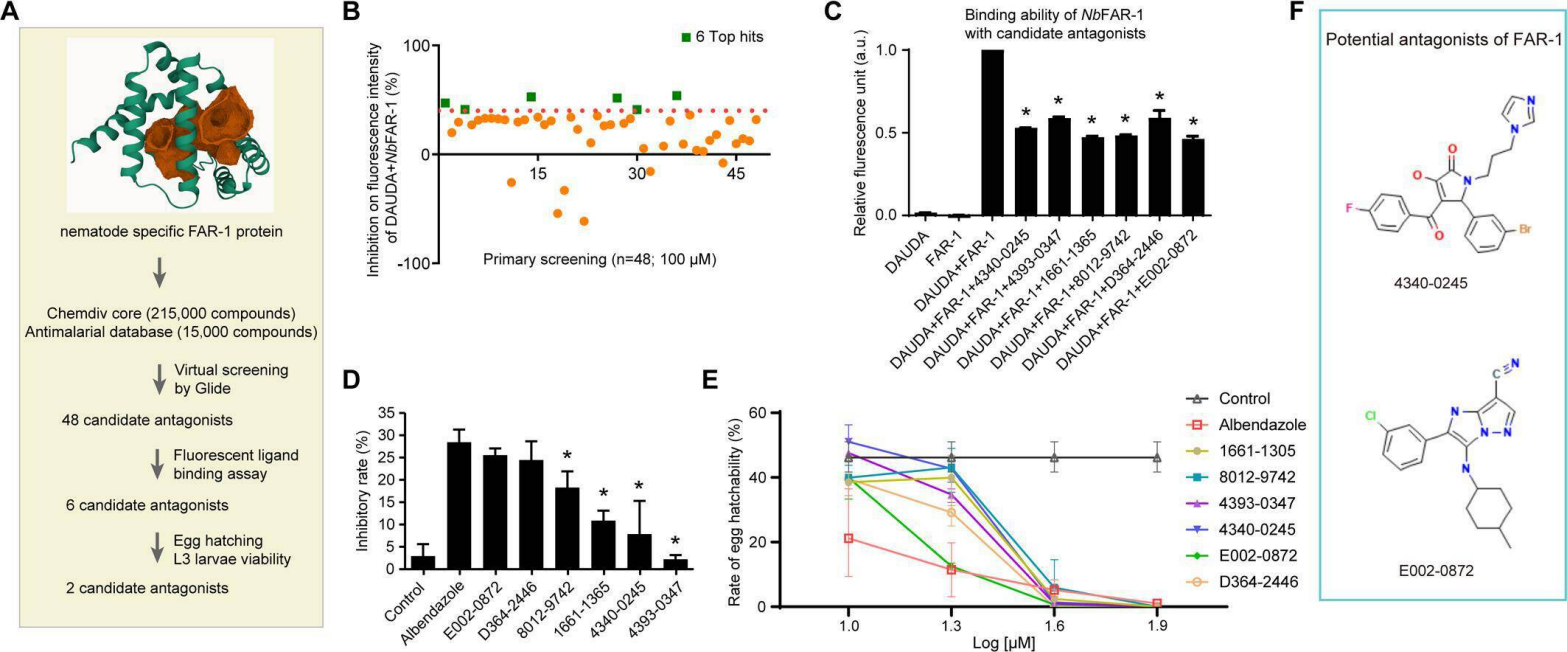
Screening the antagonists of FAR-1 with the activity of anthelmintic effect by fluorescent ligand binding assay, MTT assay, and the inhibition on egg hatching. (**A**) Schematic illustration of screening FAR-1 antagonists with the activity of anthelmintic effects. (**B**) Antagonistic effects of 48 compounds on the binding affinity of FAR-1 protein and DAUDA. (**C**) The competitive binding ability of 10-fold concentrations of 6 compounds with DAUDA to FAR-1 protein. Compared with the DAUDA+FAR-1 group. (**D**) MTT assay for six compounds on the inhibitory growth of HEK-293T cells. Compared with albendazole group. (**E**) The inhibitory effects of 6 compounds on egg hatching of *N. brasiliensis*. (**F**) The structure of compound 4340-0245 and E002-0872. *: *P* < 0.05 as determined by one-way ANOVA with Tukey’s HSD post-hoc test.

The identification of candidate antagonists of FAR-1 was primarily obtained through virtual screening and molecular docking of approximately 215,000 compounds from the ChemDiv core database and approximately 15,000 antimalarial database using Glide (Fig. 1A). The selection of 75 prioritized candidates was based on the Glide docking score. Forty-eight compounds were procured for further assessment of their binding affinity with FAR-1 protein via fluorescent ligand binding assay (Fig. 1B). Six compounds of 1661-1365, 4393-0347, D364-2446, 8012-9742, E002-0872, and 4340-0245 showed more than 40% inhibition by antagonizing the binding of *N. brasiliensis* FAR-1 protein and DAUDA (Fig. 1B and C). Furthermore, the MTT assay showed that these six compounds had inhibitory effects on the proliferation of HEK-293T cells, with inhibitory rates ranging from 2.21% to 25.53% (Fig. 1D). The inhibitory rate of the six compounds was notably lower than that of albendazole (28.44%), especially compounds 4340-0245 and 4393-0347 (Fig. 1D).

### Inhibitory effects of antagonists on egg hatching of *N. brasiliensis*

To investigate the effects of compounds on the hatching rate, the concentrations of 10 μM, 20 μM, 40 μM, and 80 μM of these compounds were added to the culture medium of the eggs. Results showed that E002-0872 had the most significant inhibitory rate of 80% on egg hatching among the six compounds at a concentration of 20 μM, a result that was analogous to that observed in the albendazole group (Fig. 1E). The remaining five compounds, at a concentration of 20 μM, showed inhibitory rates ranging from 40% to 70% (Fig. 1E). The inhibitory effects of six compounds on egg hatching were more than 95% with an increase in the concentration to 40 μM, while 4340-0245 showed the most significant concentration-dependent effect on egg hatching from 10 μM to 40 μM. Consequently, both E002-0872 and 4340-0245 were selected for further evaluation of their anthelmintic efficacy in mice (Fig. 1F).

### Anthelmintic efficacy of E002-0872 and 4340-0245 against *N. brasiliensis* infection in BALB/c mice

To evaluate the anthelmintic efficacy, E002-0872 and 4340-0245 were administered via intraperitoneal injection to infected mice at 2 h post-infection. Clinical observation showed reduced activity and rough hair in infected mice at 2 days post-infection (dpi 2), and these signs were alleviated by dpi 5 or 6. The activities of the infected mice remained relatively low in the DMSO group compared to the E002-0872 and 4340-0245 groups at dpi 5 or 6. Mice in the 15 and 20 mg/kg E002-0872 and 4340-0245 groups showed improvement in clinical signs compared to the DMSO group by dpi 8. Furthermore, the body weight of mice in the 15 mg/kg 4340-0245 group increased at dpi 8 compared to the DMSO group (Fig. S2).

Fecal egg excretion was initially observed in the DMSO group at dpi 6 and peaked at dpi 7. In the E002-0872 groups, the number of eggs per gram (EPG) at peak ovulation in the 5, 15, and 20 mg/kg E002-0872 groups was 46.15%, 34.24%, and 38.51% of that in the DMSO group, respectively; only the EPG in the 10 mg/kg group was not reduced compared to the DMSO group. In the 4340-0245 groups, the EPG at peak ovulation in the 10, 15, and 20 mg/kg groups was 60.16%, 22.24%, and 23.02% of that in the DMSO group, respectively; only the EPG in the 5 mg/kg group was not reduced compared to the DMSO group.

**Fig 2:**
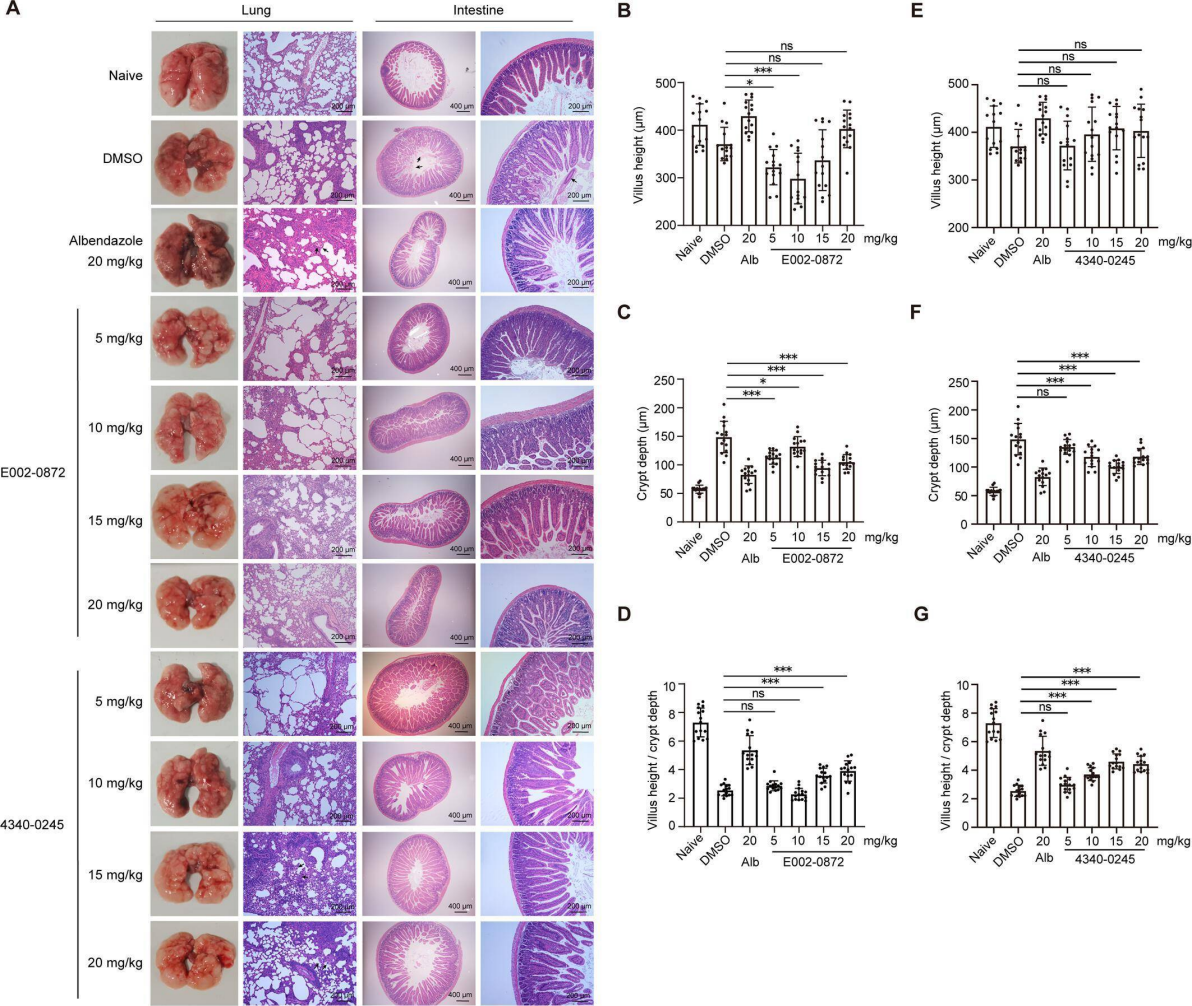
Effects of E002-0872 and 4340-0245 treatment on BALB/c mice infected with *N. brasiliensis*. (**A**) The pathological changes of lungs and intestines in infected mice treated with E002-0872 and 4340-0245 (*n* = 4). (**B–G**) The changes of intestine villus and crypt depth of infected mice treated with E002-0872 and 4340-0245 (*n* = 15). Naive: normal mice; DMSO: infected mice treated with DMSO during days 0–3; Alb: infected mice treated with albendazole; E002-0872 or 4340-0245: infected mice treated with E002-0872 or 4340-0245. Black arrow represents the worms in the lung or intestine. ns: *P* > 0.05, *: *P* < 0.05, and ***: *P* < 0.001 as determined by one-way ANOVA with Tukey’s HSD post-hoc test.

Pathological examination showed that the lungs in the DMSO group had an uneven surface, slight collapse at the edges, interstitial exudation, and hemorrhaging by dpi 8. The lung lesions in mice in the DMSO group gradually improved as the larvae migrated to the small intestine. In the albendazole group, the lungs showed severe damage characterized by a rough surface and extensive dark red hemorrhagic areas. Furthermore, HE staining showed that thickening of the alveolar walls, compensatory alveolar dilation, and the inflammatory infiltrates, particularly neutrophils and monocytes, in the bronchioles and blood vessels in the albendazole group (Fig. 2A). A small number of viable L4 larvae were also found in the lungs of infected mice in the albendazole group. In the E002-0872 groups, the lungs of infected mice in the 15 and 20 mg/kg groups had a smooth surface with only minor hemorrhagic spots at dpi 8 (Fig. 2A). Thus, lung pathology in the E002-0872 groups improved compared to the DMSO and albendazole groups, particularly in the 15 mg/kg group (Fig. 2A). In the 4340-0245 groups, lung pathology in the 15 and 20 mg/kg groups was milder than in the DMSO and albendazole groups and was accompanied by fewer hemorrhagic spots and mild swelling (Fig. 2A). L4 larvae were also found in the lungs of mice treated with 15 and 20 mg/kg 4340-0245. However, the number of L4 larvae in these groups was lower than in the albendazole group.

Analysis of intestinal pathology showed that mice in the DMSO group had swelling extending from the anterior duodenum to the anterior jejunum, accompanied by severe mucosal and tissue damage, villous rupture, and a high worm burden (Fig. 2A and B; Fig. S1). By contrast, mice in the albendazole group showed a smooth intestinal surface with no swelling in the anterior duodenum, as well as a minor reduction in the crypt-to-villus ratio compared to the naive control group (Fig. 2A through D). In the E002-0872 groups, swelling of the anterior jejunum in infected mice was significantly reduced in the 15 and 20 mg/kg groups compared to the DMSO group at dpi 8 (Fig. S1). These two groups exhibited moderate pathological changes, including slight intestinal swelling and edema of the lamina propria, as well as mild inflammatory infiltration (Fig. 2A). Furthermore, crypt depth was markedly reduced in the 15 and 20 mg/kg E002-0872 groups, resulting in a higher villus-to-crypt ratio than in the DMSO group (Fig. 2B through D). The 5 and 10 mg/kg groups showed more severe swelling and villous damage than the 15 and 20 mg/kg groups (Fig. 2B through D; Fig. S1). In the 4340-0245 groups, the 5 mg/kg group showed a higher number of adult worms in the anterior jejunum, as well as significant intestinal lesions, including swelling, villous damage, and altered crypt-to-villus ratios, which were comparable to those in the DMSO group (Fig. 2A and E through G). In contrast, the 10, 15, and 20 mg/kg groups showed intestinal surfaces with swelling and edema, but no hemorrhaging (Fig. 2A; Fig. S3). Lower crypt depth and an increased villus-to-crypt ratio were observed in the 10, 15, and 20 mg/kg groups compared to the DMSO group, particularly at the 15 mg/kg dose (Fig. 2E through G).

**Fig 3:**
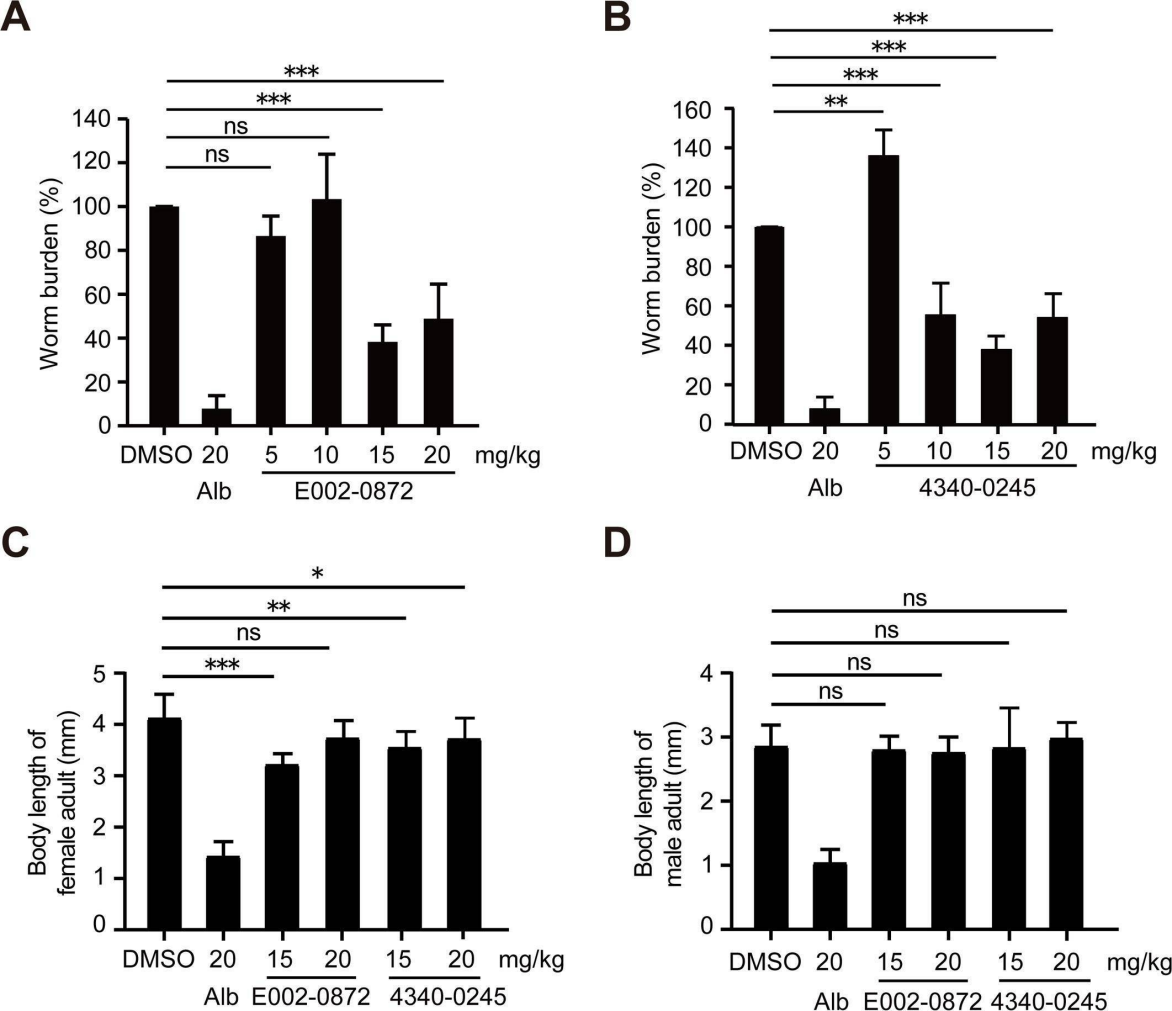
Effects of E002-0872 and 4340-0245 treatment on the development of *N. brasiliensis* in BALB/c mice. (**A and B**) The worm burden in the intestine of infected mice treated with E002-0872 and 4340-0245 (*n* = 4). (**C**) The body length of female adult of *N. brasiliensis* in the E002-0872 and 4340-0245 groups (*n* = 15). (**D**) The body length of male adult of *N. brasiliensis* in the E002-0872 and 4340-0245 groups (*n* = 15). DMSO: infected mice treated with DMSO during days 0–3; Alb: infected mice treated with albendazole; E002-0872 or 4340-0245: infected mice treated with E002-0872 or 4340-0245. ns: *P* > 0.05, *: *P* < 0.05, **: *P* < 0.01, and ***: *P* < 0.001 as determined by one-way ANOVA with Tukey’s HSD post-hoc test.

At dpi 8, the worm burden of infected mice in the 5, 15, and 20 mg/kg E002-0872 groups was 86.59%, 38.26%, and 48.84% of that in the DMSO group. No statistically significant difference in worm burden was observed in the 5 and 10 mg/kg groups compared to the DMSO group (Fig. 3A). A significant reduction in worm burden of 61.74% and 51.16% was observed in the 15 and 20 mg/kg groups, respectively (Fig. 3A). In the 4340-0245 groups, the worm burden in infected mice treated with 10, 15, and 20 mg/kg was 55.37%, 37.85%, and 54.05% of that in the DMSO group at dpi 8, corresponding to reductions of 44.63%, 62.15%, and 45.95%, respectively (Fig. 3B). A slight increase in worm load was observed in the 5 mg/kg 4340-0245 group compared to the DMSO group (Fig. 3B). This indicates that the 10, 15, and 20 mg/kg doses effectively reduced the worm load in infected mice.

The body length of female worms was 4,128.3 ± 460.4 µm in the DMSO group, 1,434.4 ± 283.8 µm in the albendazole group, 3,224.8 ± 205.2 µm in the 15 mg/kg E002-0872 group, 3,739.1 ± 335.9 µm in the 20 mg/kg E002-0872 group, 3,553.8 ± 305.5 µm in the 15 mg/kg 4340-0245 group, and 3,710.6 ± 398.1 µm in the 20 mg/kg 4340-0245 group (Fig. 3C). Female worms in the 15 mg/kg E002-0872 and 15 and 20 mg/kg 4340-0245 groups were significantly shorter than those in the DMSO group (Fig. 3C). The body length of male worms was 2,858.2 ± 328.5 µm in the DMSO group, 1,041.3 ± 207.9 µm in the albendazole group, 2,804.4 ± 211.3 µm in the 15 mg/kg E002-0872 group, 2,762.5 ± 238.8 µm in the 20 mg/kg E002-0872 group, 2,833.8 ± 617.7 µm in the 15 mg/kg 4340-0245 group, and 2,981.3 ± 244.5 µm in the 20 mg/kg 4340-0245 group (Fig. 3D). These were no significant differences compared to the DMSO group (Fig. 3D).

The antagonists E002-0872 and 4340-0245, which target the *Nb*FAR-1 protein, demonstrated an effective reduction in the burden of intestinal worms and alleviated lung and intestinal damage in BALB/c mice infected with *N. brasiliensis*. Furthermore, treatment with 4340-0245 exhibited a dose-dependent effect on the worm burden, accompanied by a slight increase in body weight in the 15 mg/kg group compared to the DMSO group, as well as the presence of some L4 larvae in the lungs of infected mice.

### Anthelmintic efficacy of 4340-0245 against *A. cantonensis* infection in BALB/c mice

**Fig 4:**
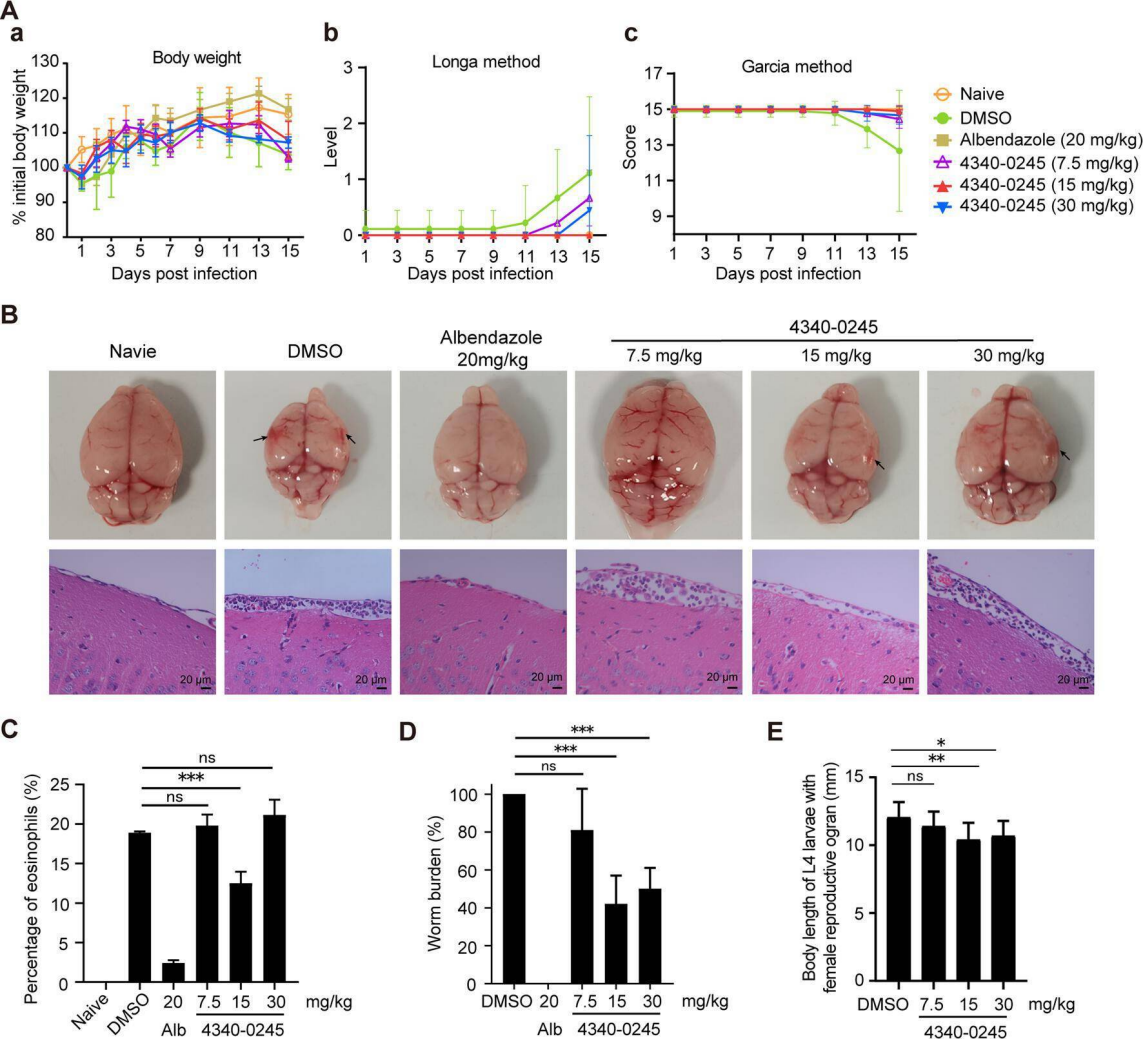
Effects of 4340-0245 treatment on BALB/c mice infected with *A. cantonensis*. (**A**a) The body weight of infected mice (*n* = 5); (**A**b) Longa method for assessing the neurological signs; (**A**c) Garcia method for assessing the neurological signs (*n* = 5). (**B**) The pathological changes of the brains in infected mice treated with 4340-0245 (*n* = 5). (**C**) The percentage of eosinophils in the brains of infected mice (*n* = 15). (**D**) The worm burden in the brain of infected mice (*n* = 5). (**E**) The body length of L4 larvae in the brain of infected mice (*n* = 10). Naive: normal mice; DMSO: infected mice treated with DMSO; Alb: infected mice treated with albendazole; 4340-0245: infected mice treated with 4340-0245. Black arrow represents hemorrhage site of mice brains. ns: *P* > 0.05, *: *P* < 0.05, **: *P* < 0.01, and ***: *P* < 0.001 as determined by one-way ANOVA with Tukey’s HSD post-hoc test.

4340-0245 was administered intraperitoneally to mice infected with *A. cantonensis* at dpi 1, at concentrations of 7.5, 15, or 30 mg/kg, once daily for 7 consecutive days. Initially, the body weight of the infected mice decreased at dpi 1, but gradually recovered from dpi 2. At dpi 15, the infected mice in the 15 mg/kg 4340-0245 group had a higher body weight than the DMSO group, but a lower body weight than the albendazole group (Fig. 4Aa). As for clinical signs, the infected mice in the DMSO group showed reduced activity, rough hair, and lethargy at dpi 9, arched back, reduced response to whisker stimulation, and limited movement and extension of the left forelimb compared to the right side at dpi 13, and some mice exhibited circling movements, head tilting, collapse with convulsion at dpi 15. The infected mice in the 7.5 mg/kg 4340-0245 group also showed reduced vitality, rough hair, and lethargy at dpi 9, followed by hunchback posture and diminished whisker response at dpi 13. The infected mice in the 15 mg/kg group showed no significant signs of neurological impairment. The infected mice in the 30 mg/kg group showed reduced vitality and mild neurological signs at dpi 13. Thus, mice in the 4340-0245 groups exhibited milder clinical signs than those in the DMSO group.

Neurological impairment was assessed using the Longa scale (0–5) and the Garcia score (1–15). At dpi 15, the average Longa level in the DMSO group was 1.11, indicating an inability to extend the unilateral anterior side (Fig. 4Ab), and the average Garcia score was 12.67, indicating mild neurological dysfunction (Fig. 4Ac). The average Longa levels at dpi 15 were 0.67, 0.00, and 0.44 in the 7.5 mg/kg, 15 mg/kg, and 30 mg/kg groups, respectively (Fig. 4Ab). The average Garcia scores at dpi 15 were 14.44, 14.89, and 14.67 in the 7.5 mg/kg, 15 mg/kg, and 30 mg/kg groups, respectively (Fig. 4Ac). Histopathological examination showed milder brain hemorrhaging and eosinophil infiltration in the meninges in the 4340-0245 groups than in the DMSO group at dpi 15 (Fig. 4B and C). However, severe hemorrhaging, meningeal thickening, and infiltration of eosinophils and inflammatory cells in the meninges were observed in the 7.5 mg/kg group, followed by the 30 mg/kg group. The least severe pathological changes were observed in the 15 mg/kg group.

The worm burden of *A. cantonensis* was significantly lower in the 4340-0245 groups than in the DMSO group. The reduction rates were 19.00%, 52.73%, and 46.36% in the 7.5, 15, and 30 mg/kg groups, respectively. No worms were detected in the brains of infected mice in the albendazole group. Antagonist 4340-0245, which targets FAR-1, had stable anthelmintic effects against nematode species.

Morphological examination revealed no significant differences in L4 larvae among groups. The body lengths of L4 larvae with female reproductive organ were 11.42 ± 1.06 mm, 10.43 ± 1.22 mm, and 10.71 ± 1.09 mm in the 7.5, 15, and 30 mg/kg 4340-0245 groups, respectively. The body lengths in the 15 and 30 mg/kg groups were shorter than the 12.08 ± 1.11 mm observed in the DMSO group. 4340-0245 effectively reduced the worm burden and impaired the development of L4 larvae with female reproductive organs in infected mice, particularly at the 15 mg/kg dose. The body length of female adult *N. brasiliensis* and L4 larvae of *A. cantonensis* with female reproductive organs was significantly affected by treatment with E002-0872 and 4340-0245, while the length of male worms remained unchanged.

The compound 4340-0245, which targets the *Nb*FAR-1 protein, demonstrated significant anthelmintic efficacy against *A. cantonensis* infection in BALB/c mice. Treatment with 4340-0245 at a dose of 15 mg/kg caused a reduction in worm burden and impaired the development of L4 larvae with the female reproductive organs, while alleviating neurological signs and reducing host tissue pathology. However, the anthelmintic effects of 4340-0245 on *N. brasiliensis* were weaker in the 20 mg/kg group than in the 15 mg/kg group, as evidenced by lower body weight, more severe clinical signs, and increased intestinal villi damage. The anthelmintic efficacy of 4340-0245 against *A. cantonensis* in the 30 mg/kg group was less than in the 15 mg/kg group. These findings suggest that the anthelmintic effects of 4340-0245 exhibit a linear, dose-dependent relationship within a specific dosage range.

### Identification and functional analysis of key residues in the FAR-1 ligand-binding pocket

**Fig 5:**
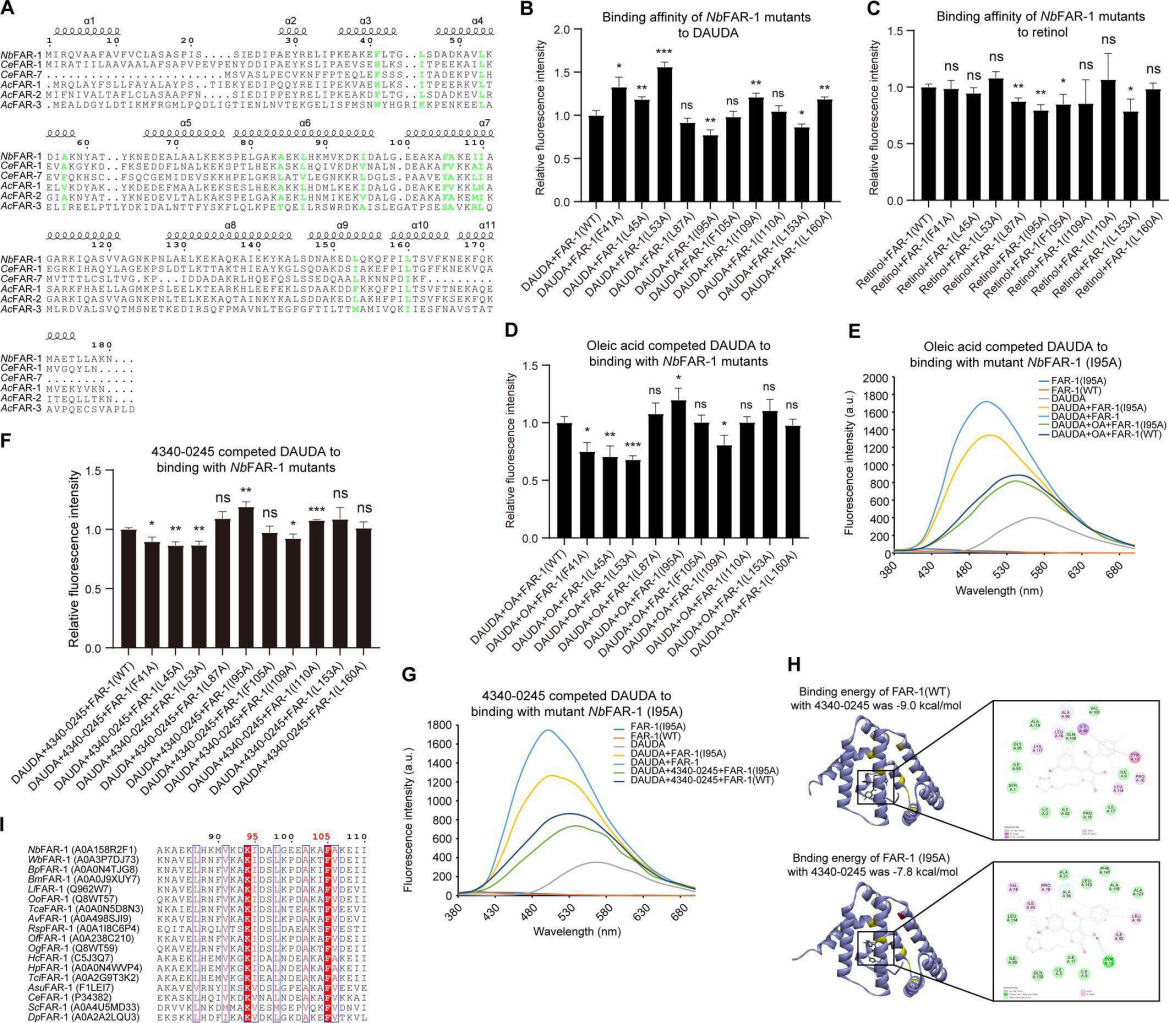
Analysis of key amino acids in the ligand binding pocket of FAR-1. (**A**) Sequence alignment of key functional sites of FAR proteins from *N. brasiliensis*, *C. elegans*, and *A. cantonensis*. The residues on the surface of ligand binding cavity (F41, L45, L53, A57, A84, L87, I95, F105, A106, I109, I110, L153, L160) were marked in bold green. (**B**) The binding ability of *Nb*FAR-1 mutants to DAUDA. (**C**) The binding ability of *Nb*FAR-1 mutants to retinol. (**D**) The oleic acid competed DAUDA to binding with *Nb*FAR-1 mutants. (**E**) The oleic acid competed DAUDA to binding with mutant *Nb*FAR-1 (I95A). (**F**) The 4340-0245 competed DAUDA to binding with *Nb*FAR-1 mutants. (**G**) The 4340-0245 competed DAUDA to binding with mutant *Nb*FAR-1 (I95A). (**H**) Comparison of the binding energy of *Nb*FAR-1 and mutant *Nb*FAR-1(I95A) with 4340-0245 by molecular docking. (**I**) Sequence alignment of I95 in FAR-1 from several nematodes. ns: *P* > 0.05, *: *P* < 0.05, **: *P* < 0.01, and ***: *P* < 0.001 as determined by one-way ANOVA with Tukey’s HSD post-hoc test.

The amino acid residues on the surface of the ligand-binding pocket of *Nb*FAR-1 protein were analyzed based on the crystal structure of *Ce*FAR-7 protein. These residues were F41, L45, L53, A57, A84, L87, I95, F105, A106, I109, I110, L153, and L160 (Fig. 5A). To assess the functional roles of these residues, alanine scanning mutagenesis was employed to replace them with alanine residues. Prokaryotic expression and purification were employed to obtain the wild-type *Nb*FAR-1 protein and 10 mutants: FAR-1(F41A), FAR-1(L45A), FAR-1(L53A), FAR-1(L87A), FAR-1(I95A), FAR-1(F105A), FAR-1(I109A), FAR-1(I110A), FAR-1(L153A), and FAR-1(L160A) (Fig. S4).

The binding affinity of DAUDA, retinol, oleic acid, and 4340-0245 to *Nb*FAR-1 was evaluated in both the wild-type and the mutants. The binding affinity of DAUDA increased compared to FAR-1(WT) for the mutants F41A, L45A, L53A, I109A, and L160A (Fig. 5B; Fig. S5A and B), whereas it decreased for the mutants I95A and L153A (Fig. 5B; Fig. S5A and B). Retinol showed decreased binding affinity to the mutants L87A, I95A, F105A, and L153A compared to FAR-1(WT) (Fig. 5C; Fig. S5C and D). The competitive binding affinity of oleic acid increased for the mutants F41A, L45A, L53A, and I109A (Fig. 5D; Fig. S6), but decreased for the mutant I95A (Fig. 5D and E). The competitive binding affinity of 4340-0245 to the mutants F41A, L45A, L53A, and I109A increased compared to FAR-1(WT), while it decreased for the mutants I95A and I110A (Fig. 5F and G; Fig. S7). Antagonist 4340-0245 exhibited similar binding characteristics to *Nb*FAR-1 as fatty acid. These results indicated that I95 was a critical residue for binding ligands. Molecular docking results also showed that the binding energy of the FAR-1(I95A) mutant with ligands increased due to the alanine mutation (Fig. 5H; Table S2).

### Acute toxicity analyses of 4340-0245 on BALB/c mice

**Fig 6:**
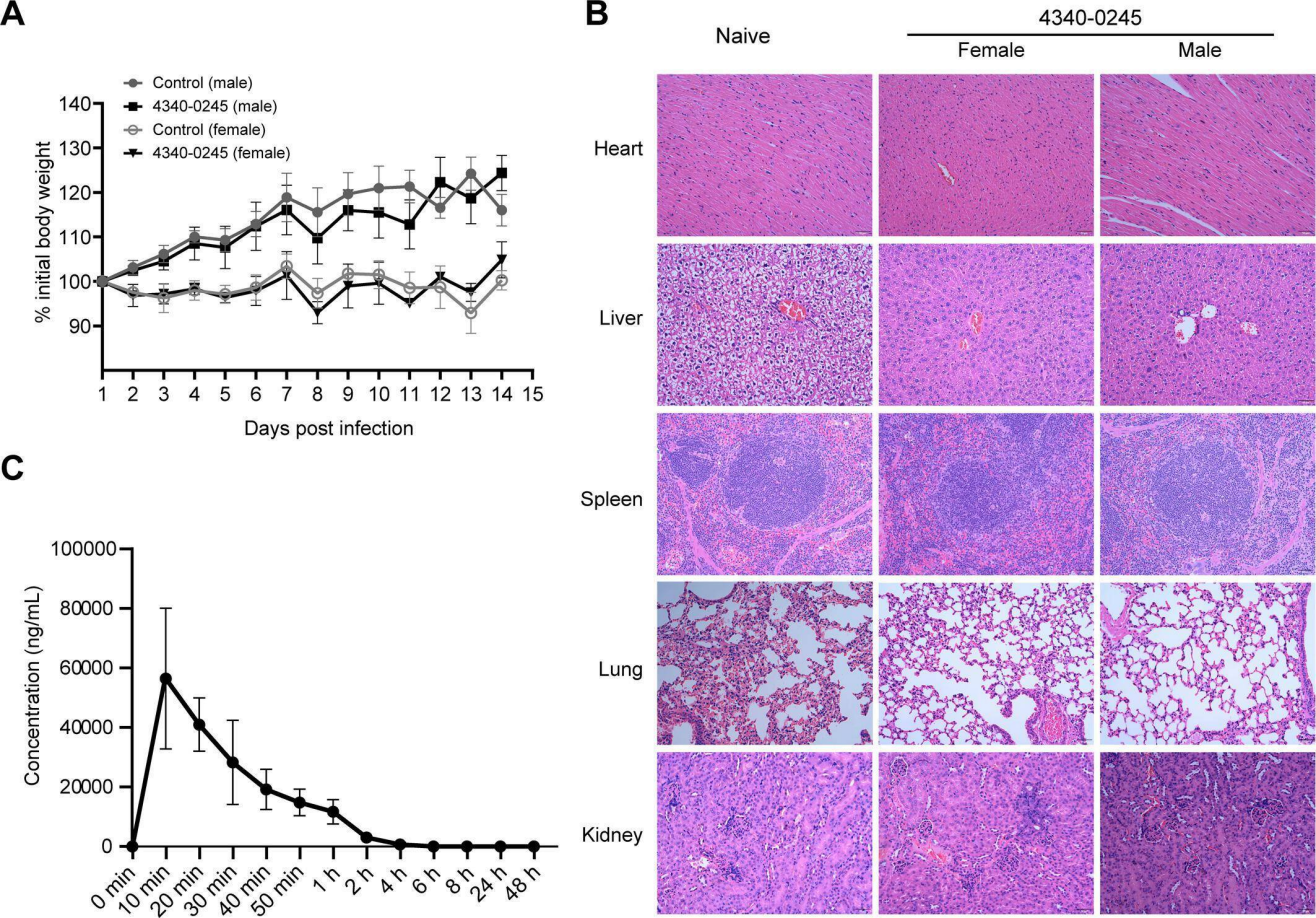
The effects and metabolism of 4340-0245 to the BALB/c mice by intraperitoneal injection. (**A**) The changes of body weight of BALB/c mice treated with 50 mg/kg 4340-0245 (female *n* = 6, male *n* = 6). (**B**) H&E staining observation of organs in mice treated with 50 mg/kg 4340-0245, Bar = 50 µm (female *n* = 6, male *n* = 6). (**C**) Time curve of plasma concentration in BALB/c mice treated with 15 mg/kg 4340-0245 (*n* = 5).

The intraperitoneal administration of 4340-0245 at a dose of 50 mg/kg to both male and female mice did not result in the fatalities. No changes were observed in appearance, behavior, mental state, appetite, fecal consistency, and fur condition. Additionally, no abnormal secretions were detected in the nose, eyes, or mouth. As for the body weight, male and female mice in the 4340-0245 group exhibited a steady increase comparable to the control group, respectively (Fig. 6A). HE staining showed no exudate, hemorrhaging, or inflammatory cell infiltration in the major organs of mice treated with 4340-0245 (Fig. 6B). A single administration of a high dosage of 50 mg/kg of 4340-0245 did not exhibit significant acute toxicity in mice, which was consistent with the low inhibitory rate of 4340-0245 on HEK-293T cell growth. Thus, no significant adverse effects were observed in the BALB/c mice following administration of 4340-0245.

### Pharmacokinetic properties of 4340-0245 in BALB/c mice

Accurate measurement of the concentration of 4340-0245 in mouse plasma is essential for pharmacokinetic analysis and for supporting the efficacy and safety of the compound. A validated LC-MS/MS method was established to quantify 4340-0245 in mouse plasma (Fig. S8). The retention time of 4340-0245 was 2.31 min with no interference from endogenous plasma components (Fig. S8B). A linear standard curve (1–100,000 ng/mL) was established for quantification (Fig. S9), with a lower limit of quantification (LLOQ) of 10 ng/mL. The method was assessed for precision, extraction recovery, and stability. The relative standard deviations (RSDs) for precision were 6.3%, 8.4%, and 2.3% for the low (20 ng/mL), medium (1,000 ng/mL), and high (80,000 ng/mL) quality control samples, respectively; all were within ±10% (Table S3). Extraction recovery ranged from 67.8% to 80.6%, with RSD within ±10% (Table S3). 4340-0245 was stable in plasma under the following conditions: storage at room temperature for 6 and 24 h, three freeze-thaw cycles, and storage at −20°C for 15 days, with RSD within ±10% (Table S4).

Following a single intraperitoneal injection of 15 mg/kg of 4340-0245 in BALB/c mice, plasma concentrations of 4340-0245 were measured at various time points (Table S5). The observed pharmacokinetics in the mouse plasma included the time to reach maximum concentration (*t*_max_ = ~10 min), peak concentration (*C*_max_ = 56,473.1 ng/mL), and area under the concentration-time curve (AUC_(0-t)_ = ~2,393,220.1 min·ng/mL, AUC_(0-∞)_ = ~2,395,647.1 min·ng/mL), the terminal half-life (*t*_1/2_ = 430.0 min), and the clearance rate (CL/F = 6.26 mL/min·kg) (Table S6). Following injection, 4340-0245 was rapidly absorbed and metabolized into the plasma, reaching a maximum concentration.

## DISCUSSION

The limited number of available anthelmintic drugs, coupled with their adverse effects and the increasing threat of drug resistance, highlights the urgent need to identify novel therapeutic targets for helminth control. The nematode-specific FAR-1 protein has emerged as a highly promising candidate for antagonist screening. The expression level of *far-1* genes is high across developmental stages; moreover, *far-1* genes share high sequence identity in key animal and human parasitic nematodes from Clade V (7). This study aims to identify novel FAR-1 antagonists from the large compound databases, which could then be optimized into effective anthelmintic drug candidates and pave the way for novel therapies against parasitic nematodes.

The drug screening process is well-established, enabling rapid and efficient identification of potential antagonists. In this study, the employment of virtual screening, competitive binding assays, and MTT assay has identified the potential antagonists of FAR-1 protein from extensive compound libraries, thereby facilitating the subsequent elucidation of anthelmintic effects. Thus, nematode FAR-1 protein serves as an excellent target for high-throughput screening, offering a robust platform for the rapid discovery of antagonists from compound libraries.

Nematode eggs are transparent and can be easily cultured, with hatching into L1 larvae occurring within 24 h under laboratory conditions. This characteristic facilitates rapid screening of antagonists by assessing their egg-hatching rates. Among six compounds, we chose two compounds with distinct inhibitory effects on the egg hatching. E002-0872 exhibited the highest inhibitory rate of 80% in 20 μM concentration, while 4340-0245 showed a concentration-dependent effect on the inhibition of egg hatching, with inhibitory rates of 49% (10 μM), 57% (20 μM), and 99% (40 μM) (Fig. 1E; Table S7). *In vivo* anthelmintic efficacy of E002-0872 and 4340-0245 on *N. brasiliensis* in a mouse model showed that both compounds had a closed reduction rate in the worm burden of 61.74% and 62.15% in the 15 mg/kg group (Table S7), thereby showing their strong anthelmintic potential. However, only 4340-0245 exhibited a dose-dependent effect; moreover, 4340-0245 treatment caused a slight increase of the body weight of infected mice in the 15 mg/kg group compared to the DMSO group, and some L4 larvae were found in the lung and could not enter the intestine of infected mice. Furthermore, the reduction rate of the worm burden for *A. cantonensis* was 52.73% in the 15 mg/kg 4340-0245 group (Table S7). Antagonist 4340-0245, targeting FAR-1, had stable anthelmintic effects against different nematodes. Thus, analysis of the anthelmintic effects of antagonists should combine egg hatching *in vitro* and larvae development in *vivo*.

The anthelmintic effects of compounds E002-0872 and 4340-0245 on *N. brasiliensis* were weaker in the 20 mg/kg groups than in the 15 mg/kg group, as evidenced by lower body weight, more severe clinical signs, and increased damage to the intestinal villi. A reduction in anthelmintic efficacy of compound 4340-0245 on *A. cantonensis* was also observed in the 30 mg/kg group compared to the 15 mg/kg group. These findings suggest that the anthelmintic effects of 4340-0245 exhibit a linear, dose-dependent relationship within a specific dosage range. However, exceeding this range can lead to decreased anthelmintic effects. A similar pattern had been seen with aspirin: its effect was linearly dose-dependent up to a certain point, but once that threshold was exceeded its metabolic pathways could become saturated, leading to slower rises in plasma concentration, a prolonged half-life, and a cascade of toxic side effects (23, 24). It is hypothesized that the exacerbation of symptoms seen at a high dosage of 4340-0245 may be due to compound accumulation in the body and consequently increasing the risk of toxicity.

The body length of female adult of *N. brasiliensis* and L4 larvae with female reproductive organs of *A. cantonensis* was significantly affected by the treatment with E002-0872 and 4340-0245 (Table S7), while the length of male worms remained unchanged. These findings suggest that female worms were more sensitive to treatment with both antagonists than male worms. Moreover, analysis of the expression pattern during nematode development revealed the higher expression levels of *far-1* in female adults than in male adults of both *N. brasiliensis* and *A. cantonensis* (7, 11).

The clinical signs and pathological changes in mice are crucial indicators for evaluating the anthelmintic effects of compounds. Treatments with antagonist could alleviate clinical signs and mitigate intestinal villus damages in mice infected with *N. brasiliensis* and reduce neurological damages in mice infected with *A. cantonensis*. In the life cycle, L4 larvae of *N. brasiliensis* migrate from host lungs to the intestines, then the lung lesions of mice in the DMSO group gradually improved as the larvae migrated to the small intestine. A small number of L4 larvae of *N. brasiliensis* were found in the lungs in the albendazole group. The L4 larvae were also detected in the lungs of mice treated with 15 mg/kg and 20 mg/kg of 4340-0245 though in lower numbers than observed in the albendazole group. Moreover, no L4 larvae of *N. brasiliensis* were found in the lungs of mice treated with E002-0872. This is primarily because albendazole had the significant anthelmintic effects on *N. brasiliensis* larvae in the lungs, killing the larvae or preventing the migration of some larvae with reduced vitality to the intestine. The more retained larvae in the albendazole group caused more extensive pulmonary lesions than in the mice treated with 4340-0245, whereas the histopathological changes observed in the 4340-0245 and E002-0872 groups did not differ significantly. Overall, 4340-0245 exhibited superior anthelmintic efficacy against nematode infection compared to E002-0872.

The FAR protein is characterized by its conserved three-dimensional structure, which is rich in alpha helices. The amino acid I95 within the ligand-binding pocket plays a crucial role in the binding of ligands. Sequence alignment analysis showed that most nematode FAR-1 proteins possess a conserved I95 residue; however, in certain nematodes, this position is occupied by valine (V95) (Fig. 5I). Both isoleucine and valine are alpha amino acids, featuring an amino group (-NH_2_) and a carboxyl group (-COOH) attached to the same alpha carbon atom. These functional groups endow isoleucine and valine amphiphilic properties, enabling them to exhibit cationic properties in acidic environments and anionic properties in alkaline environments. They exist as zwitterionic (amphoteric) ions at their isoelectric point. Furthermore, isoleucine and valine are classified as branched-chain amino acids due to the branching of their side chains. Isoleucine has an isopropyl side chain, while valine has an isobutyl side chain. This branching imparts similar steric hindrance effects within protein structures, thereby influencing protein folding and stability.

Safety is a crucial factor in the development of new drugs (25). A high dosage of 50 mg/kg 4340-0245 at a single administration does not exhibit significant acute toxicity in mice, which was consistent with the low inhibitory rate of 4340-0245 on the growth of HEK-293T cells. Thus, no significant adverse effects were observed in the BALB/c mice following administration of 4340-0245. Following intraperitoneal injection of 4340-0245 at a dosage of 15 mg/kg, the compound was rapidly absorbed and metabolized into the mouse plasma and reached a maximum concentration, suggesting that 4340-0245 can effectively prevent L3 larvae from migrating to the lungs via the bloodstream. In contrast, the pharmacokinetic parameters of albendazole are well documented. It typically exhibits a shorter half-life and lower *C*_max_, which may contribute to its stronger anthelmintic efficacy (26). These differences could potentially account for the weaker anthelmintic effects of 4340-0245 than albendazole.

### Conclusion

The antagonists E002-0872 and 4340-0245, which target the *Nb*FAR-1 protein, were found to be highly effective in reducing the worm load of *N. brasiliensis* by over 60% in BALB/c mice. Furthermore, 4340-0245 effectively reduced the worm burden of *A. cantonensis* in the brains of infected mice by over 50%. Antagonists could impact the development of female worms and mitigate lesions and tissue damage in infected mice. Antagonist 4340-0245 had no significant toxic effects on HEK-293T cells or BALB/c mice; however, improving the absorption and metabolism of 4340-0245 in BALB/c mice could enhance its anthelmintic effects. Overall, our findings established FAR-1 as a promising anthelmintic target and suggested that optimizing the structure of its antagonists could enhance therapeutic efficacy.

## SUPPLEMENTAL MATERIAL

Supplemental material aac.01226-25-s0001.docx Fig. S1 to S9; Tables S1 to S7.

ASM does not own the copyrights to Supplemental Material that may be linked to, or accessed through, an article. The authors have granted ASM a non-exclusive, world-wide license to publish the Supplemental Material files. Please contact the corresponding author directly for reuse.
