# Supplementary material for: Structure-guided screening of FAR-1 antagonists with multi-stage anthelmintic activity
Source: Antimicrob Agents Chemother. 2026 Apr 15;70(5):e01226-25. doi: 10.1128/aac.01226-25 (PMC13148050; doi:10.1128/aac.01226-25)
Supplement: Supplemental material — Fig. S1 to S9; Tables S1 to S7. [file aac.01226-25-s0001.docx]

**Supplementary Figures and Tables:**


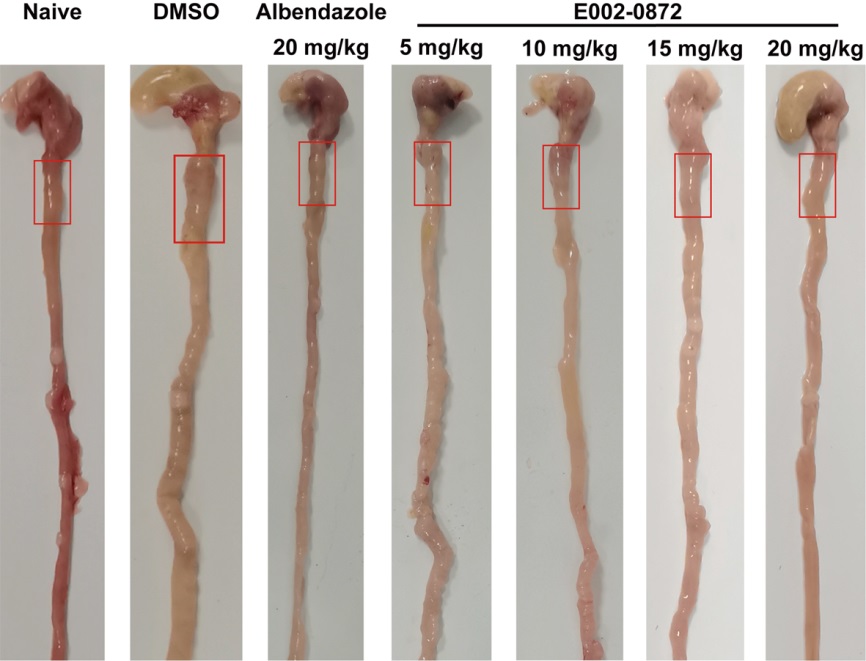


Supplementary Figure S1 Treatment with E002-0872 on the intestine of BALB/c mice infected with *N. brasiliensis*. The red box means the aggregation of *N. brasiliensis* in the anterior of the duodenum, resulting in edema and hemorrhage.


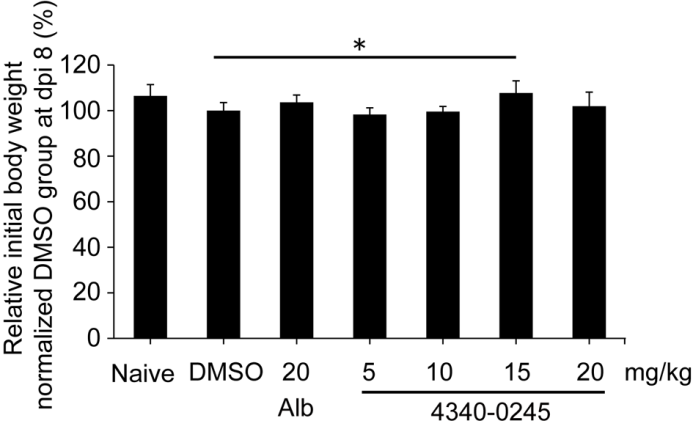


Supplementary Figure S2 The body weight of BALB/c mice infected with *N. brasiliensis* following treatment with 4340-0245 at dpi 8.


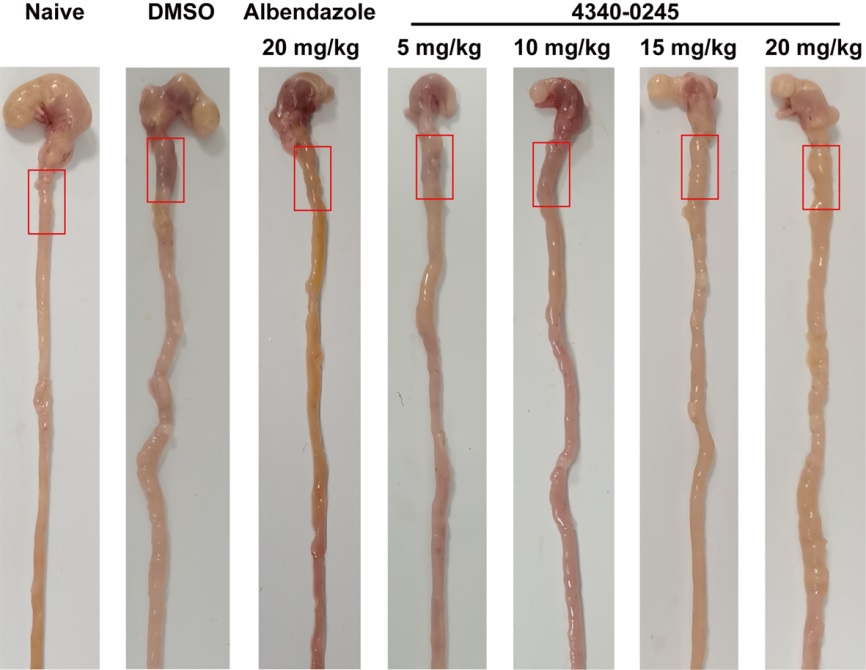


Supplementary Figure S3 Treatment with 4340-0245 on the intestine of BALB/c mice infected with *N. brasiliensis*. The red box indicates the anterior segment of the duodenum parasitized with *N. brasiliensis.*


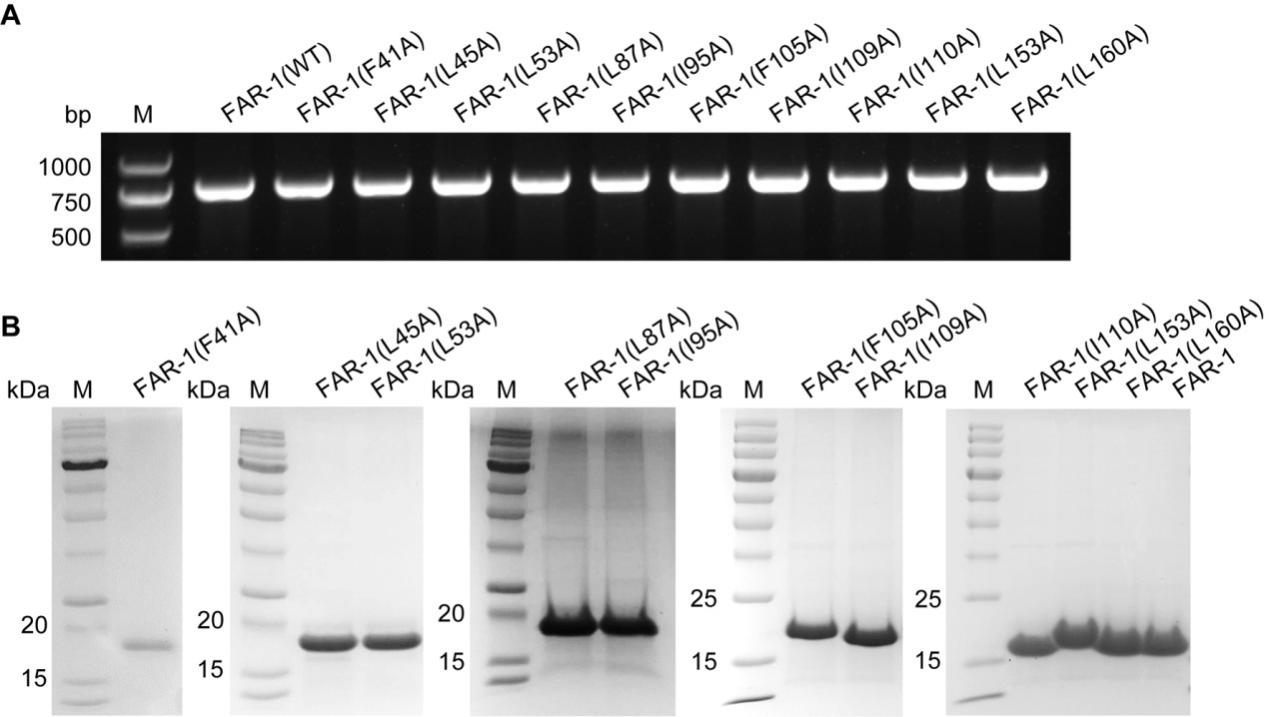


Supplementary Figure S4 Construction of point mutation of *far-1* gene and expression and purification of FAR-1 mutants.

1. Introduced point mutation in *far-1* gene by PCR amplification; (B) Expression and purification of FAR-1 mutants.


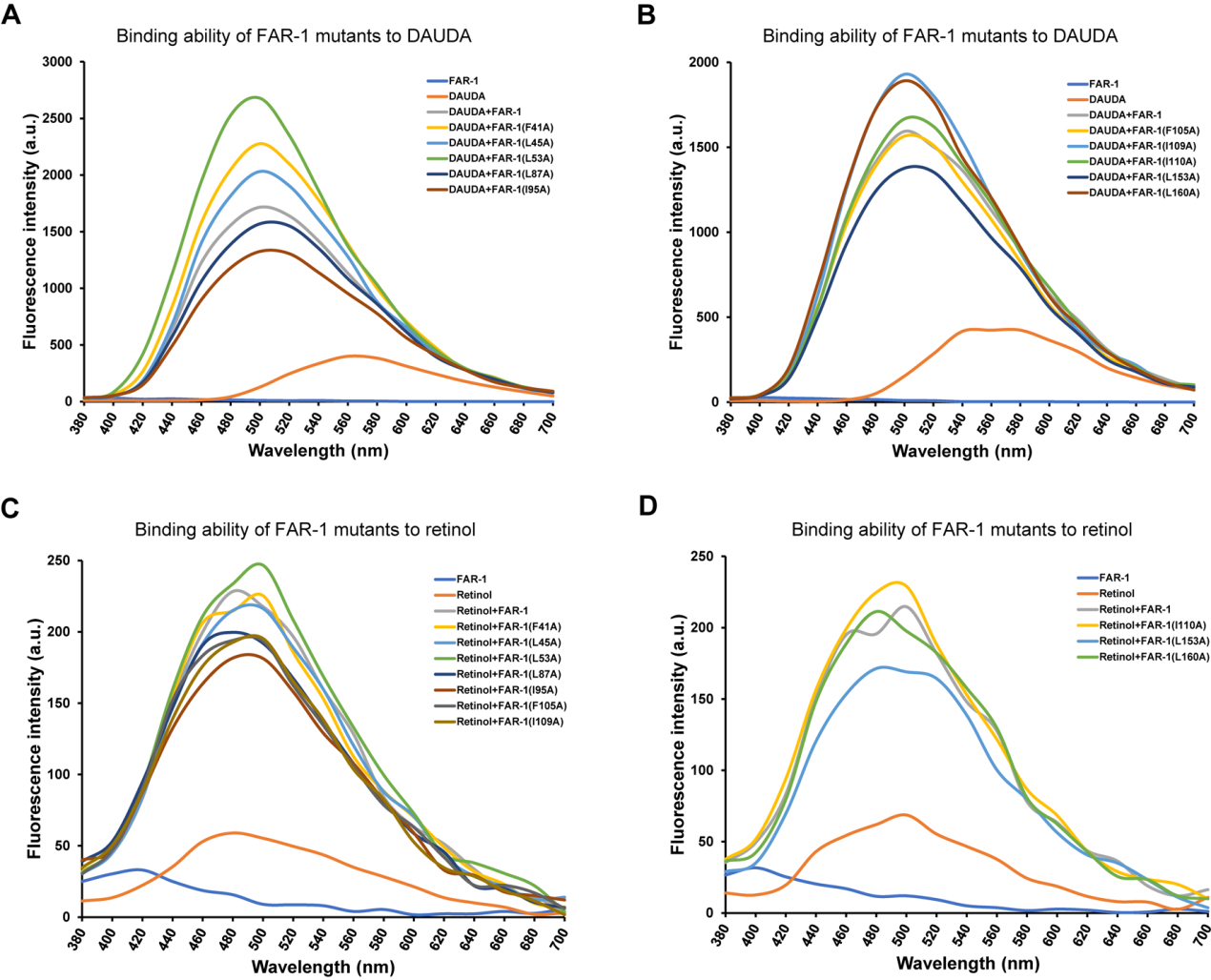


Supplementary Figure S5 The binding affinity of FAR-1 mutants with DAUDA and retinol by fluorescent ligand binding assay.

1. B) The binding ability of FAR-1 mutants to DAUDA. (C-D) The binding ability of FAR-1 mutants to retinol.


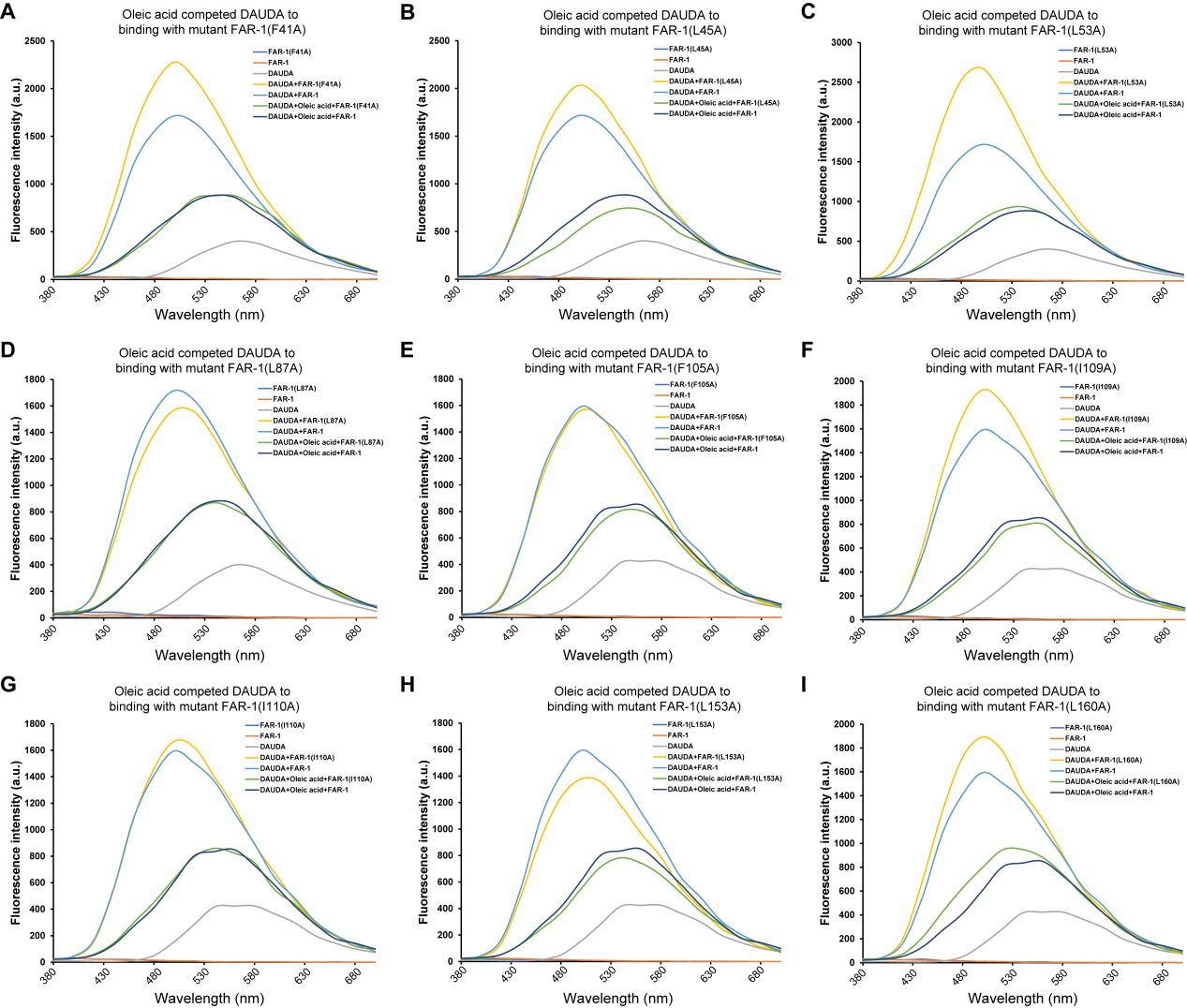


Supplementary Figure S6 The binding affinity of FAR-1 mutants with oleic acid by fluorescent ligand competition binding assay.

(A-I) Oleic acid competed with DAUDA for binding to FAR-1 mutants (F41A, L45A, L53A, L87A, F105A, I109A, I110A, L153A, and L160A).


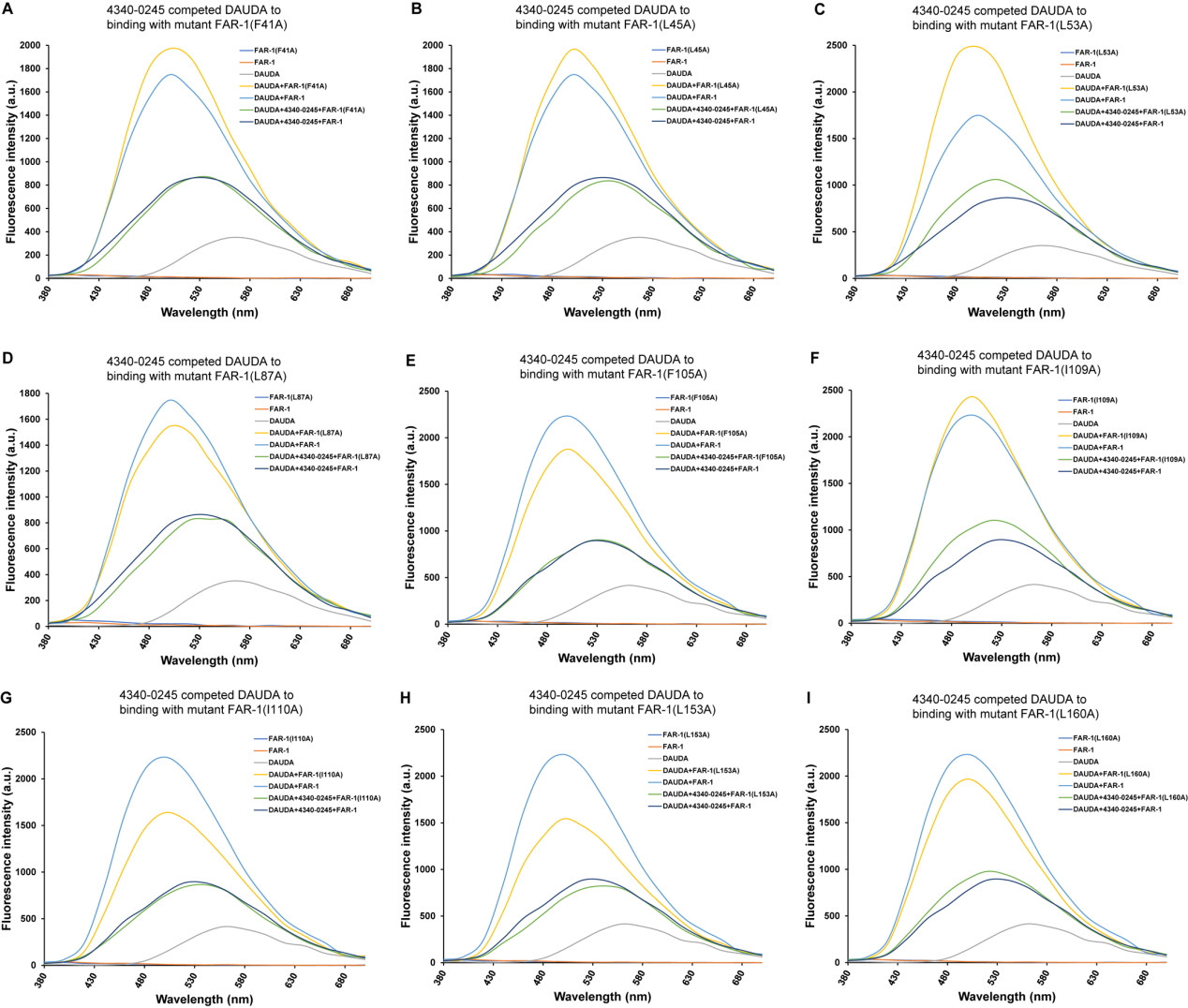


Supplementary Figure S7 The binding affinity of FAR-1 mutants with 4340-0245 by fluorescent ligand competition binding assay.

(A-I) 4340-0245 competed with DAUDA for binding to FAR-1 mutants (F41A, L45A, L53A, L87A, F105A, I109A, I110A, L153A, and L160A).


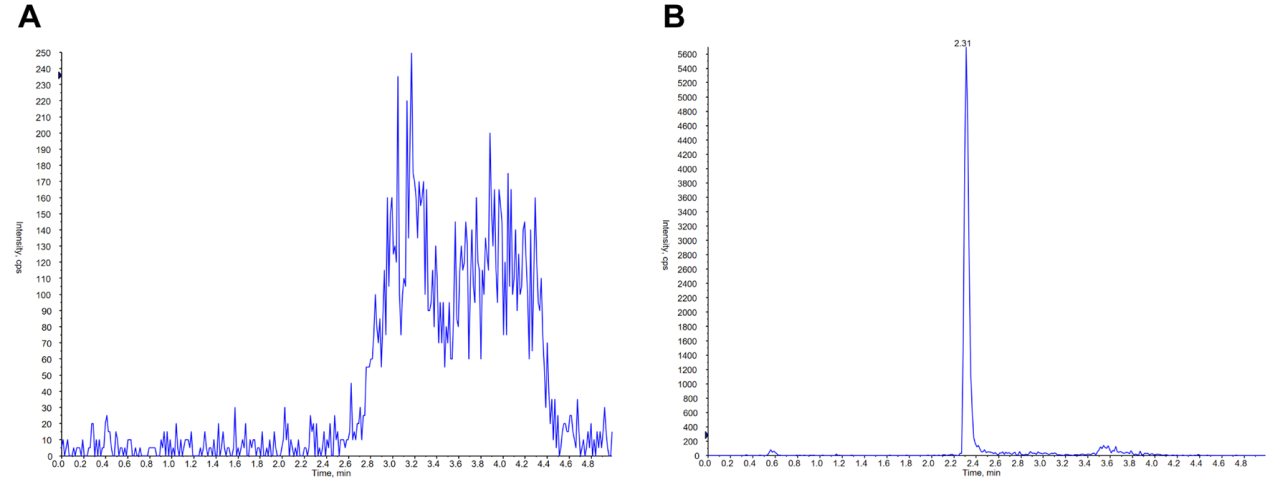


Supplementary Figure S8 Chromatograms of mouse plasma and plasma containing 4340-0245.

1. Chromatogram of mouse plasma; (B) Chromatogram of mouse plasma containing 100 ng/mL 4340-0245.


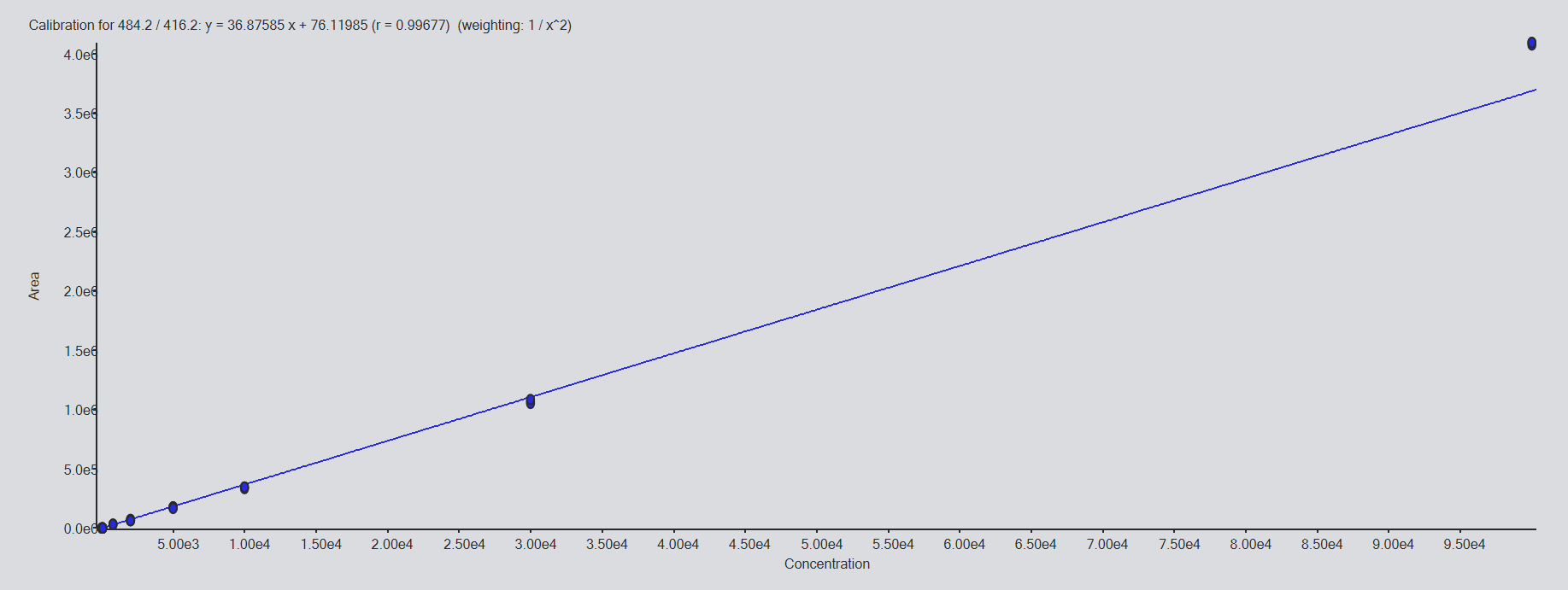


Supplementary Figure S9 A linear standard curve of 4340-0245 in BALB/c mice plasma detected by LC-MS/MS.

Supplementary Table S1 Primer sequences for constructing mutants of *Nb*FAR-1

| Name | Primer sequences |
| --- | --- |
| F41A-F | AGGCCAAGGAGGCCCTCACCGGACTGTCCGAT |
| F41A-R | TCCGGTGAGGGCCTCCTTGGCCTCCTTGGGGAT |
| L45A-F | TTCCTCACCGGAGCGTCCGATGCTGACAAGGCT |
| L45A-R | GCATCGGACGCTCCGGTGAGGAACTCCTTGG |
| L53A-F | ACAAGGCTGTGGCGAAGGACATCGCCAAGAAC |
| L53A-R | ATGTCCTTCGCCACAGCCTTGTCAGCATCGG |
| L87A-F | GCCGAGAAGGCCCACAAAATGGTTAAGGACAAG |
| L87A-R | CATTTTGTGGGCCTTCTCGGCCTTAGCTCCAAG |
| I95A-F | TAAGGACAAGGCCGACGCTCTCGGAGAGGAGGC |
| I95A-R | GAGAGCGTCGGCCTTGTCCTTAACCATTTTGTGG |
| F105A-F | CTAAGGCTGCCGCCAAGGAGATCATCGCTG |
| F105A-R | TCCTTGGCGGCAGCCTTAGCCTCCTCTCC |
| I109A-F | GCCAAGGAGGCCATCGCTGGAGCTCGCAA |
| I109A-R | TCCAGCGATGGCCTCCTTGGCGAAAGCCT |
| I110A-F | AAGGAGATCGCCGCTGGAGCTCGCAAGAT |
| I110A-R | AGCTCCAGCGGCGATCTCCTTGGCGAAAGC |
| L153A-F | AAGGAAGACGCCCAGAAGCAGTTCCCCATCCTC |
| L153A-R | GCTTCTGGGCGTCTTCCTTGGCGTTGTCGGA |
| L160A-F | TTCCCCATCGCCACCTCCGTCTTCAAGAACGA |
| L160A-R | CGGAGGTGGCGATGGGGAACTGCTTCTGGA |

The line means the mutated base.

Supplementary Table S2 The binding energy of mutant FAR-1(I95A) and DAUDA, retinol, oleic acid and 4340-0245

| Ligand | FAR-1 | Binding energy (kcal/mol) |
| --- | --- | --- |
| DAUDA | FAR-1(WT) | -7.7 |
|  | FAR-1(I95A) | -6.2 |
| Retinol | FAR-1(WT) | -8.0 |
|  | FAR-1(I95A) | -7.2 |
| Oleic acid | FAR-1(WT) | -6.3 |
|  | FAR-1(I95A) | -5.4 |
| 4340-0245 | FAR-1(WT) | -9.0 |
|  | FAR-1(I95A) | -7.8 |

Supplementary Table S3 The precision and recovery rate of 4340-0245 in the plasma of BALB/c mice

|  | | Concentration of 4340-0245 in mouse plasma (ng/mL) | | |
| --- | --- | --- | --- | --- |
|  |  | 15 | 800 | 80000 |
| Precision | Detect value (ng/mL) | 13.8±0.9 | 815.5±68.2 | 88192±2058 |
|  | RSD (%) | 6.3 | 8.4 | 2.3 |
| Recovery rate | Detect value (%) | 67.8±6.1 | 79.6±4.3 | 80.6±3.6 |
|  | RSD (%) | 8.9 | 5.4 | 4.4 |

Note: value means average ± SD.

Supplementary Table S4 The stability of 4340-0245 in the mouse plasma with distinct treatments

| Treatment | 4340-0245 | 15 (ng/mL) | 80000 (ng/mL) |
| --- | --- | --- | --- |
| Room temperature for 6 h | Measured plasma concentration (ng/mL) | 14.3±0.7 | 89444±1600 |
|  | RSD (%) | 4.9 | 1.8 |
|  | Accuracy（%） | 95.3 | 111.8 |
| Room temperature for 24 h | Measured plasma concentration (ng/mL) | 13.9±0.9 | 89006±3099 |
|  | Relative standard deviation (%) | 6.5 | 3.5 |
|  | Accuracy（%） | 92.6 | 111.3 |
| 3 cycles of freeze-thaw treatment | Measured plasma concentration (ng/mL) | 14.1±1.1 | 89919±2020 |
|  | Relative standard deviation (%) | 7.9 | 2.2 |
|  | Accuracy（%） | 93.9 | 112.4 |
| -20°C for 15 days | Measured plasma concentration (ng/mL) | 14.7±1.1 | 88423±2018 |
|  | Relative standard deviation (%) | 7.7 | 2.3 |
|  | Accuracy（%） | 98.2 | 110.5 |

Note: value means average ± SD.

Supplementary Table S5 Plasma concentration of 15 mg/kg 4340-0245 in mice by intraperitoneal injection

| Time | Plasma concentration (ng/mL) |
| --- | --- |
| 0 min | 0.00±0.00 |
| 10 min | 56473.06±23678.75 |
| 20 min | 40997.95±8965.30 |
| 30 min | 28280.35±14148.96 |
| 40 min | 19223.73±6732.93 |
| 50 min | 14820.36±4440.71 |
| 1 h | 11706.36±4112.13 |
| 2 h | 3025.85±432.63 |
| 4 h | 680.99±668.12 |
| 6 h | 42.84±15.17 |
| 8 h | 28.74±10.22 |
| 24 h | 7.46±4.92 |
| 48 h | 2.61±3.34 |

Note: value means average±SD.

Supplementary Table S6 Pharmacokinetic parameters of 4340-0245 in BALB/c mice

| Pharmacokinetic parameters | 4340-0245 in BALB/c mice |
| --- | --- |
| C_max_（ng/mL） | 61300.2±17449.7 |
| T_max_（min） | 12.0±4.5 |
| t_1/2_（min） | 430.0±170.5 |
| AUC_(0-t)_（min·ng/mL） | 2393220.1±461446.6 |
| AUC_(0-∞)_（min·ng/mL） | 2394564.8±461821.9 |
| CL/F（mL/min·kg） | 6.46±1.3 |

Note: value means average ± SD.

Supplementary Table S7 The anthelmintic activities of FAR-1 antagonists on *N. brasiliensis* and *A. cantonensis*

| Nematode | Effects | 4340-0245 | E002-0872 |
| --- | --- | --- | --- |
| *N. brasiliensis* | Egg hatching rate  (*in vitro* culture) | 20 µM: 42.71% ± 3.76% | 20 µM: 13.19% ± 1.59% |
|  | Fecal EPG at peak ovulation | 15 mg/kg: 22.24% of that in the DMSO group | 15 mg/kg: 34.24% of that in the DMSO group |
|  | Worm burden | 15 mg/kg: 37.85% of that in the DMSO group | 15 mg/kg: 38.26% of that in the DMSO group |
|  | Female length | 15 mg/kg: 3553.8 ± 305.5 μm  DMSO: 4128.3 ± 460.4 μm | 15 mg/kg: 3224.8 ± 205.2 μm  DMSO: 4128.3 ± 460.4 μm |
| *A. cantonensis* | Worm burden | 15 mg/kg: 47.27% of that in the DMSO group | -- |
|  | L4 larvae with female reproductive organ | 15 mg/kg: 10.43 ± 1.22 mm  DMSO: 12.08 ± 1.11 mm | -- |
|  | PK parameters | 15 mg/kg:  *t*_max_ = ~10 min  *C*_max_ = 56,473.1 ng/mL  AUC_(0-t)_ = ~2,393,220.1 min·ng/mL  AUC_(0-∞)_ = ~2,395,647.1 min·ng/mL  *t*_1/2_ = 430.0 min | -- |

-- means no detection.
